# Suicide behaviour and Problematic Internet Use

**DOI:** 10.1192/j.eurpsy.2022.220

**Published:** 2022-09-01

**Authors:** J. Ortuño-Sierra, B. Lucas Molina, A. Gutiérrez, R. Aritio Solana, A. Pérez-Albéniz, E. Fonseca Pedrero

**Affiliations:** 1 University of La Rioja, Educational Sciences, Logroño, Spain; 2 University of Valencia, Psychology, Logroño, Spain; 3 Univerity of La Rioja, Educational Sciences, Logrono, Spain; 4 Univerity of La Rioja, Educational Sciences, Logroño, Spain

**Keywords:** mental health, adolescence, suicide behaviour, problematic Internet use

## Abstract

**Introduction:**

The use of internet among children and adolescent has risen in the last decade. In addition, suicide is the second cause of death among adolescents. Previous research have indicated the relation between Problematic Internet Use (PIU) and different mental health problems. Nonetheless there is a lack of studies analyzing the relation between suicide behaviour and PIU

**Objectives:**

The main objective of the present work was to analyze the relation between Problematic Internet Use and suicide behaviour and depression in adolescents

**Methods:**

A total of 1036 adolescents (450 males) were randomly selected. Mean age was 15,21 (SD = 1,23). The Adolescent Behavioural Suicide Scale SENTIA, The Reynolds Adolescent Depression Scale Short Form (RADS-SF), and The Compulsive Internet Use Scale (CIUS) were used. A Manova was performed with two groups (risk and non-risk to PIU) as independent variables and suicide and depression scores as dependent variables

**Results:**

The results revealed a statiscally signifficant association between PIU and both depression and suicide behaviour (λ = 0.245, F(2,81,000) = 15.549, P ≤ 0.001, η² = 0.116). In particular, adolescents at a higher risk for PIU obtained higher scores on suicide behaviours and depression.

**Conclusions:**

Results found in the present study reveal that adolescents have moderate prevalence rates for PIU. Also adolescents at risk for PIU with a total of more than 3 hour sof internet use everyday were at a higher risk for suicide. Prevention strategies should be devote to intervene in internet use as it maybe a variable affecting suicide behaviour.

**Disclosure:**

No significant relationships.

